# The extended farm effect: The milk protein β-lactoglobulin in stable dust protects against allergies 

**DOI:** 10.5414/ALX02246E

**Published:** 2022-03-29

**Authors:** Hanna Mayerhofer, Katharina Zednik, Isabella Pali-Schöll

**Affiliations:** 1 Interuniversity Messerli Research Institute of the University of Veterinary Medicine Vienna and Medical University Vienna, and; 2 Institute of Pathophysiology and Allergy Research, Medical University Vienna, Vienna, Austria

**Keywords:** allergy prevention, β-lactoglobulin, farm effect, lipocalin, zinc

## Abstract

Background: The allergy- and asthma-protective farm effect is mediated by numerous factors. Especially dust from cattle stables and raw cow’s milk show beneficial properties, suggesting a bovine protein to be involved. As a major milk protein and member of the lipocalin family, β-lactoglobulin (BLG) binds small, hydrophobic ligands and thereby modulates the immune response. Empty BLG promotes allergy development, whereas BLG in association with ligands shows allergy-preventive as well as allergy-reducing effects in vivo and in vitro. Results: BLG has been identified as a major protein in stable dust (therein bound to zinc) as well as in the air around cattle stables. This association with zinc favors an allergy-protective immune profile. Conclusion: Its immune-modulating, allergy-protective characteristics together with its presence in raw cow’s milk as well as in stable dust and ambient air render BLG an essential contributor to the farm effect.

## Introduction 

Children growing up on farms have a reduced risk of developing asthma and allergies by up to 50% [1]. This phenomenon is described by the so-called farm effect, which has been demonstrated in numerous studies. Over the years, several potential components mediating this effect have been revealed, however, raw cow’s milk as well as stable dust proved to be particularly effective in protecting against atopic diseases. In addition, other factors such as genetic predisposition, husbandry of (domestic) animals (Mayerhofer et al., Allergologie 2022, under review) and contact with certain microbes also play an important role [2]. 

## Asthma and farms 

Asthma prevalence has steadily increased worldwide in recent years, presumably due to urbanization and, subsequently, the decline of closeness to farms. Comparing children growing up on farms with children growing up in cities, farm children show a significantly lower asthma incidence and prevalence. There was also a dose-response effect, as children who do not live on farms but have regular contact with stables and barns were found to have intermediate asthma prevalence [3]. This study additionally analyzed the protection by different types of farms and identified the keeping and breeding of dairy cows and cows for meat production in combination with grain and corn farming as especially effective. Children’s exposure to farm features showed that i) contact with cattle, ii) living near cattle, iii) contact with straw as well as iv) exposure to manure had beneficial effects and lowered asthma prevalence in children. 

## Prenatal influences 

Not only the stay of children on farms influences the prevalence of asthma and hay fever. Prenatal exposure to microbial products and the mother’s consumption of non-pasteurized milk also shows a positive effect. Postnatally, the child should have constant contact with the protective factors to ensure optimal protection [4]. Studies in mouse models confirmed these observations and showed that the protective effect by microbial extracts in stable dust is dependent on toll-like receptors (TLR) [5], whereas contact of pregnant mice with live pathogens no longer contributed to diaplacental protection against asthma. This implies that sustained exposure of the maternal immune system to benign microbial stimuli is a key mechanism. These benign microbial stimuli trigger TLR without inducing inflammation that would be caused by pathogenic stimuli [6]. In addition, infants whose mothers are exposed to a bacteria-rich environment before birth exhibited increased expression of TLR-2, TLR-4, and CD14 genes, which are key components of innate immune defense [7]. Exposure to moderate amounts of lipopolysaccharides attenuates signaling pathways involved in the synthesis of allergy-associated cytokines via TLR activation, thereby activating the production of Th1 cytokines by dendritic cells [8]. Ultimately, this counteracts allergy. Consistent with this finding, a more rapid shift from a Th2-skewed, atopic immune status to a more balanced and mature Th1 immune profile can be observed in farm-raised children postnatally [6]. In addition, raw milk consumption in pregnant women has been shown to promote demethylation of forkhead-box protein 3 (Foxp3) in the unborn, thereby reducing the risk of asthma in the child [9]. 

Contacts with farm animals and with cats during pregnancy showed equally beneficial effects: they increased levels of secretory immunoglobulin A (sIgA) in breast milk, which reduced the prevalence of atopic dermatitis in children [10]. Accordingly, increased levels of sIgA and transforming growth factor-β (TGF-β) in breast milk as well as prolonged breastfeeding protected children against wheezing [11]. 

## Bacterial load as benefit 

In addition, the microbiome as well as its products are often discussed as key protective factors against asthma and allergies. Endotoxins (bacterial lipopolysaccharides) from farm environments have been shown to contribute to protection against asthma. Closer studies of dust from children’s beds revealed an inverse relationship between endotoxin levels and asthma prevalence in these children, confirming endotoxins to be important determinants of the farm effect [12]. In this regard, the ubiquitin-modifying enzyme A20, alias tumor necrosis factor α-induced protein 3 (TNFAIP3) in lung epithelial cells appears to mediate the effect of endotoxin [13]. 

Children growing up on farms are exposed to a broader range of bacteria than children who do not grow up on a farm. This greater diversity is in turn indirectly proportional to asthma [14]. It seems that not only the diversity of bacterial species, but also the microbiota composition of household dust on farms is relevant. A recent study showed that the more similar the microbiome of urban households became to farm households, the lower the risk of asthma in children from urban households was. In contrast to urban households, bovine bacteria of the orders *Bacteroidales*, *Clostridiales* as well as *Lactobacillales* were much more frequent in farm households [15]. 

## The role of the gut 

The gut microbiome and its metabolites are involved in asthma prevention. Fatty acids are the most abundant bacterial metabolites in the gut. One study examined the difference between short-chain fatty acids in the gut of farm children and those children who do not live on a farm [16]. Farm children had higher levels of a short-chain fatty acid, valeric acid, which correlated with a lower incidence of eczema. In addition, the number of siblings and the keeping of pets (specifically dogs and cats) also influenced the amount of valeric acid. It was concluded that an increased number of short-chain fatty acids is the result of a more mature as well as more complex gut microbiome in farm children and that especially increased occurrence of valeric acid contributes to the protective farm effect. 

## The raw milk effect 

The protective farm effect also includes the consumption of raw milk, of which the protective efficacy has been shown to be independent of the stable [17]. 

This epidemiological observation was confirmed in a mouse model: mice pretreated with raw milk developed a lower allergic response after sensitization with a house dust mite allergen than those animals that were not pretreated with raw milk [18, 19]. 

The effect of raw milk is mediated by the following components: IgG, miRNA, fatty acids, oligosaccharides, microbes, and most importantly whey proteins [2]. The latter were also identified for the first time in the GABRIELA study as key proteins of raw milk: the proteins α-lactalbumin, β-lactoglobulin (BLG) as well as bovine serum albumin were found to reduce asthma prevalence [1]. For the protein BLG, our research group was able to show that together with its ligands like iron-siderophores, vitamin A and D, or zinc, can protect against allergic immune reactions [20, 21, 22, 23, 24, 25]. 

Although raw milk and its proteins are less likely to cause hypersensitivity than processed products, and even actively protect against asthma and allergy development [26], the incidence of raw milk consumption declines. A possible cause is the increasing urbanization and thus greater geographical distances from rural areas. Moreover, raw milk consumption cannot be recommended due to the potential presence of pathogenic germs, such as *Salmonella*, *Listeria*, or enterohemorrhagic *Escherichia coli* [27, 28]. 

## Influence of milk processing 

To prevent diseases caused by the above-mentioned pathogens, commercial milk is processed by heating before distribution. Therefore, processing of milk also serves the purpose of extending shelf life. However, heating to ~ 75 °C for 15 – 30 seconds not only kills the undesirable microorganisms but also alters valuable components of milk that contribute to allergy protection [1, 29]. One study showed that temperatures as low as 65 °C lead to a structural change of milk proteins and thus to a loss of protection, since immunologically active milk proteins already denature at this temperature. These are heat-sensitive proteins such as BLG and α-lactalbumin. Ultra-heat-treated milk contains only very small amounts of intact proteins, and simultaneously the allergy protection is apparently lost. In addition, heat-induced aggregation of milk proteins was observed starting at 75 °C, which was getting more intense at higher temperatures (above 80 °C) [18]. 

To circumvent this problem, new approaches are currently being tested, such as minimal processing of milk. The ongoing MARTHA study is currently investigating potential differences between consumption of minimally processed milk and ultra-heat-treated milk from the supermarket [18]. The protective effect is analyzed in children aged 6 months to 3 years with respect to asthma prevalence. 

## BLG as a key factor 

The fact that raw cow’s milk consumption and independently farm contact mediates the farm effect implies the involvement of proteins of animal origin, specifically those associated with cattle. One important bovine protein is BLG. As a central whey protein, BLG (or *Bos d 5*) is considered to be the main allergen in cow’s milk and accounts for ~ 12% of the total protein [30]. BLG belongs to the protein family of lipocalins, which possess an intramolecular pocket to incorporate small, hydrophobic ligands. Vitamins and their metabolites (retinoic acid, vitamin D3) as well as hormones (adrenaline) and iron-binding siderophores (catecholates) act as potential ligands [31]. 

## Allergy prevention via BLG 

The loading of this pocket of BLG has a decisive influence on the development of allergy: unloaded BLG, so-called apo-BLG, promotes the increase of CD4+ T cells as well as the expression of Th2 cytokines and subsequently allergies and inflammation, whereas BLG loaded with ligands (holo-BLG) suppresses CD4+ T helper cells in vivo and in vitro, and thus has immunosuppressive effects [21, 32, 33]. The allergy-preventive effect of BLG was also demonstrated on the unrelated birch pollen allergen Bet v 1 in animal models [22]. In agreement with these results, PBMC from birch pollen-allergic patients stimulated with holo-BLG developed a lower Th2 immune response than the apo-BLG-treated cells [24, 39]. 

Human lipocalins, such as lipocalin-2 (LCN-2), alias neutrophil gelatinase-associated lipocalin (NGAL), also have a similar effect on the immune system. LCN-2 is primarily expressed in the lung and intestine, and its loading with iron-siderophores also determines the subsequent immune response [34, 35]. In humans, increased LCN-2 is considered a biomarker for tumor as well as renal diseases [36]. Accordingly, allergic patients have significantly lower serum LCN-2 levels [37]. 

## BLG is also present in stable dust 

**Figure 1:**
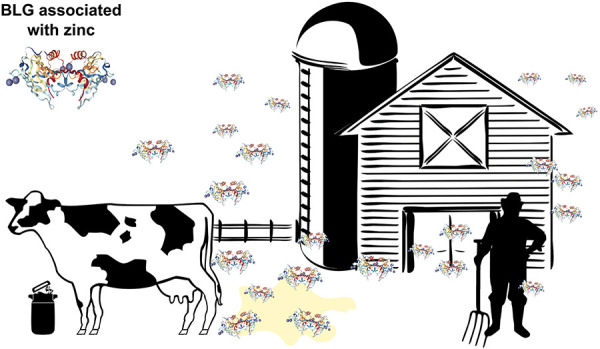
BLG in combination with zinc is a central protein in the cattle stable dust and in ambient air, into which it is aerosolized after excretion via bovine urine. Its allergy-preventive and allergy-reducing potency in vitro renders it an important component of the farm effect. *Sources: Vector graphics: cow, stable: Clker-Free-Vector-Images@Pixabay; Farmer: mohamed_hassan@Pixabay; Urine: PlumePloume@Pixabay; Milk jug: Tom Seidel@pixabay; Source Protein Structure: RSCB Protein Data Bank (https://www.rcsb.org/structure/4LZV).*

As a cattle-specific protein, the presence of BLG was also verified on cattle farms. BLG was identified as a central protein in ambient air and in stable dust (Figure 1). Therein, it was associated with zinc in its holo-form [25]. Urine from both female and male cattle served as the primary source of BLG. Thus, the protein accumulates in dust after excretion and subsequently enters the air as an aerosol, where it was detected in decreasing concentrations within a radius of up to 290 m around the stable. The function of BLG for the cattle themselves has not been elucidated yet, but it is thought to play a role in the innate immune system (as in humans). 

In more detailed investigations, BLG could also be found in the dust of farm households [25], which indicates that people in the household and around the farm who do not have direct contact with stables also benefit from the protective effect. 

## The effect of BLG association with zinc 

To investigate the effect of BLG-zinc on the cellular system, PBMC of healthy donors were stimulated with BLG or BLG-zinc. BLG associated with zinc resulted in lower proliferation of CD4+ and CD8+ T cells and promoted a Th1 cytokine milieu, which ultimately counteracts allergy development [25]. 

The receptor responsible for cellular uptake of BLG has not been elucidated to date, but the lipocalin-interacting membrane receptor (LIMR) is a promising candidate [38]. 

## BLG in practical application 

After a thorough investigation of the preclinical data, BLG and its ligands (including zinc) were tested for their allergy-reducing effect in the form of a lozenge. In double-blind, placebo-controlled studies in patients allergic to birch pollen [39] and house dust mite [24], this remedy for a supplementary balanced diet efficiently reduced allergic symptoms. Ongoing studies will now clarify the mechanism of action of this lozenge and the cellular uptake kinetics of BLG and its ligands. 

## Conclusion 

BLG in combination with zinc is a key protein in bovine stable dust and, together with zinc and other binding partners, shows allergy-preventive and allergy-reducing activity in vitro and in vivo. Thus, BLG with its binding partners is a central contributor to the farm effect. 

## Funding 

None. 

## Conflict of interest 

The authors have no conflict of interest with respect to this publication.
